# A study protocol for individualized prognostic counselling in the palliative phase

**DOI:** 10.1186/s12904-025-01647-z

**Published:** 2025-01-10

**Authors:** Boyd Noël van den Besselaar, A. Sewnaik, M. C. Dorr, A. Hoesseini, J. A. Hardillo, R. J. Baatenburg de Jong, M.P.J. Offerman

**Affiliations:** Department of Otorhinolaryngology and Head and Neck Surgery, Erasmus MC Cancer Institute, University Medical Centre Rotterdam, Dr. Molewaterplein 40, Rotterdam, 3015 GD The Netherlands

**Keywords:** Head and neck squamous cell cancer, Prognostic model, Palliative care, Proactive care planning, Prognostic counselling

## Abstract

**Background:**

Head and neck squamous cell cancer (HNSCC) has a poor prognosis, with approximately 25–30% of patients transitioning into the palliative phase at some point. The length of this phase is relatively short, with a median duration of five months. Patients in this stage often have increased prognostic information needs. Unfortunately, predicting individual life expectancy in this phase is particularly challenging, as physicians and patients tend to overestimate survival. To address this issue, we developed the prognostic model OncologIQ Palliative based on user preferences. In this study, we now aim to assess the clinical impact of utilizing this model during counselling.

**Methods:**

This study will employ both quantitative and qualitative approaches. The primary outcome is decisional conflict and satisfaction with the decision-making process after counselling without (cohort 1) and with (cohort 2) OncologIQ Palliative. Therefore, a prospective sequential cohort study will be conducted. Secondary outcomes include the amount of palliative treatment, overall survival rates, and quality of life. These measurements will be collected after the intervention. Additionally, patients’ perspectives on the decision-making process and proactive care planning, including end-of-life discussions, will be explored through interviews.

**Discussion:**

By offering more personalized prognostic information for HNSCC patients in the palliative phase, we anticipate a shift towards more patient-centred counselling. This approach can facilitate enhanced end-of-life discussions and better proactive care planning. Patients may experience reduced decisional conflict, feel better prepared for what’s coming, and find assistance in their decision-making process. This could potentially lead to fewer palliative treatments. Overall, these aspects can contribute to a better quality of life and quality of care for HNSCC patients in the last phase of their lives.

**Trial Registration:**

This study was registered November 18, 2024, on ClinicalTrials.gov: NCT06699316.

**Supplementary Information:**

The online version contains supplementary material available at 10.1186/s12904-025-01647-z.

## Background

### Background and rationale

Worldwide, head and neck squamous cell cancer (HNSCC) accounts for more than 700.000 new cases and 350.000 deaths annually [1]. In general, HNSCC has a poor prognosis, with over 40% of patients dying as a result of their disease [2]. Consequently, a large proportion of patients eventually reach the palliative phase [3], which begins when cure is no longer possible or when curative treatment is refused. In this stage, patients may experience specific problems such as dyspnoea, pain, psychosocial problems and the risk of a potential blowout [4–9]. 

The survival of patients with incurable HNSCC is relatively short, with a median of five months, although individual survival can range from one week to two years [10, 11]. Given this short length of the palliative phase, it is of key importance that patients have the best information available, such as accurate prognoses on quality of life (QoL) and life expectancy. Discussing prognosis and talking about death early in the disease trajectory enables patients to identify goals and make well considered end-of-life choices, which could contribute to a better QoL and quality of death [12, 13]. Studies have shown that patients who discuss end-of-life care with their physician are less likely to receive burdensome treatments, like chemotherapy, and more likely to receive hospice care [14, 15]. Some research even suggest that palliative or hospice care does not reduce survival and may actually prolong it [16, 17]. 

Despite patients’ desire for more detailed prognostic information [18–21], accurate individual life expectancy predictions for palliative HNSCC patients remain lacking. Physicians mainly rely on general estimates and their own experience, and their estimates are often inaccurate as they tend to overestimate survival [11, 22–26]. Additionally, physicians often feel uncomfortable and reluctant to discuss prognosis in the palliative phase, partly due to concerns about potentially being proved inaccurate [27]. Lack of accurate individual prognoses can result in suboptimal use of palliative and end-of-life care, and therefore, reduced QoL.

Besides meeting patients’ information needs, providing prognostic information in the palliative phase can influence their end-of-life choices. Decision making around treatment is challenging for both doctors and patients, often involving uncertainty and potential decisional conflict [28]. Personalized survival estimates allow for more realistic end-of-life discussions, enabling optimization of palliative care planning and impacting treatment decisions and participation in clinical trials [14, 15, 26]. 

Over the past years, a prognostic model named ‘OncologIQ’ for patients in the curative phase has been developed, validated, and recently updated with new prognostic factors [23, 29–31]. Unlike traditional survival rates based solely on TNM-classification, OncologIQ incorporates individual patient characteristics (such as age and the Adult Comorbidity Evaluation 27) to provide a more personalized estimate of the remaining life-span. Such prognostic models could aid physicians in delivering more accurate predictions of prognosis for individual patients. A recent clinical study demonstrated that counselling using OncologIQ improved decision-making [32]. However, until recently, a comprehensive prognostic model for palliative HNSCC patients was lacking.

To address this gap, we initiated the development of OncologIQ Palliative to predict individual survival for head and neck squamous cell carcinoma (HNSCC) patients in the palliative phase [33]. Recently, we validated, designed, and visualized it in an online dashboard [34, 35]. Our next step is to clinically test the effect of prognostic counselling using this model. We will investigate the impact of counselling patients without and with the prognostic model on decisional conflict and satisfaction with the decision-making process. Secondary aims include measuring satisfaction with proactive care planning after counselling with the prognostic model, as well as examining differences in terms of palliative treatment, overall survival rates, and QoL between cohorts.

### Methods/design

We will set up a single centre prospective sequential trial with two cohorts in a palliative HNSCC patient population. This study design is chosen in favour of a randomized controlled trial, because potential bias can be avoided as doctors develop a learning curve while working with the palliative model and could change their way of counselling. We aim to investigate the difference in effect of (prognostic) counselling without and with the use of OncologIQ Palliative by obtaining outcomes on decisional conflict, the amount of palliative treatment, palliative sedation or euthanasia, overall survival and QoL. The two cohorts in this study will be divided based on the type of prognostic counselling received. Cohort 1 will consist of patients who receive prognostic counselling without the model, reflecting the current clinical practice. Cohort 2 will comprise patients who receive additional prognostic counselling with the model. Additionally, we will conduct a qualitative evaluation through interviews among patients in cohort 2. This evaluation aims to investigate satisfaction with proactive care planning, including end-of-life discussions on preferred place of death and wishes for palliative sedation and euthanasia, following counselling with the use of OncologIQ Palliative.

Our hypothesis is that sharing personalized prognostic information on life expectancy with HNSCC patients in the palliative phase will empower patients throughout the decision-making process from the start of the palliative trajectory till death. More specifically providing this individualized prognostic information with the help of our prognostic model will lead to less decisional conflict and more satisfaction with the decision-making process. Additionally, we anticipate greater satisfaction with proactive care planning, including end-of-life discussions on preferred place of death and wishes for palliative sedation and euthanasia. Furthermore, when patients are optimally informed, they may choose palliative treatments less often, and it could lead to a higher QoL.

### Settings and participants

#### Settings

This prospective sequential cohort study will be situated at the Erasmus MC in Rotterdam, the Netherlands.

#### Participants and eligibility criteria

To be eligible for participation in the clinical study and qualitative evaluation, individuals must meet specific criteria. These include patients with HNSCC in the palliative phase and tumours situated in the oral cavity, oropharynx, nasopharynx, hypopharynx, supraglottic larynx, glottis larynx and unknown primary. Additionally, participants must have adequate understanding of the Dutch language and be aged 18 years or older. Exclusion criteria encompass individuals who are illiterate, have insufficient proficiency in the Dutch language, or lack decision-making capacity to consider their own treatment choices due to factors such as mental incapacitation.

### Sample size and power calculations

#### Clinical study

The power calculation is based on the primary outcome: decisional conflict. This implies that with an expected improvement on the decisional conflict scale of 8 points (out of 100), with a standard deviation of 17 points 79 patients per group are needed to find a statistical significant difference (α: 0.05; power: 0.80) [36]. Therefore we will include 160 patients in total, meaning *n* = 80 in each cohort. To our knowledge, there are no studies among palliative head and neck cancer patients who measure decisional conflict. Therefore, the study we used for estimating the standard deviation remains a ‘second best’ guess.

### Qualitative evaluation

In cohort 2 we will use a purposive sampling method to select patients, striving for a reflection of the patient group in terms of sex, age and tumour location. There are no additional selection criteria for participating in the interview. We cannot calculate a specific sample size due to the qualitative design. Therefore, we will continue including patients upon data saturation is achieved.

### Recruitment and study procedures

#### Clinical study

Patients who are transitioning into a palliative HNSCC trajectory will be consecutively approached to participate in the study. The investigator will start by screening eligible patients after their first or follow-up visit. Once the patient has been discussed in the RWHHT, the researcher will check whether the palliative trajectory is confirmed.

In cohort 1, during the visit in which the palliative trajectory is discussed, the physician will provide counselling without the individualized prognostic model. As part of standard care, the patient will then visit the nurse practitioner immediately after their appointment with the physician. During this visit, the nurse practitioner or the investigator will inform patients about the study and present the subject information sheet. Patients will be asked to participate, and the informed consent (IC) form will be collected digitally.

In cohort 2, during the visit in which the palliative trajectory is discussed, the physician will inform the patient about the option to receive additional individualized prognostic counselling on their life-expectancy using OncologIQ Palliative. Considering that most patients have prognostic inquiries when discussing the palliative trajectory (*‘Doctor*,* how long will I live?’)*, we would like to offer them the model’s prognostic information directly or in a follow-up consultation, according to their preference. We believe it is unethical to let them wait for several days if they want to receive more prognostic information right away. If patients opt not to receive their individually calculated prognosis, they will be counselled without the model and offered general information about prognosis. However, if patients prefer not to receive any information on prognosis, their preference will be respected. As part of standard care, the patient will directly visit the nurse practitioner after their appointment with the physician. During this visit, the nurse practitioner or the investigator will provide the patient with additional oral information and the subject information sheet. We will only inform patients if they have received counselling with the model. If these patients wish to continue participating, we will ask them to sign the IC digitally.

To measure decisional conflict, patients will be asked to complete the Decisional Conflict Scale (DCS) questionnaire two weeks after the consult in which they received counselling. This can be done either online or by telephone. Therefore, the DCS is integrated into Healthcare Monitor, our electronic system for measuring patient-reported outcome measures (PROMs), enabling us to digitally send it to patients [37]. Data on QoL will be obtained using the EORTC QLQ-C15-PAL questionnaire, which is already included in Healthcare Monitor and is also part of our standard care for all patients with HNSCC in the palliative phase. The patient and tumour characteristics, such as smoking behaviour, ACE-27, and WHO-performance status will be collected at baseline. Additionally, data on palliative treatment, palliative sedation or euthanasia and overall survival will be gathered from the electronic patient files (EPFs).

### Qualitative evaluation

In cohort 2, we will also evaluate satisfaction with the decision-making process and proactive care planning (including end-of-life discussions) through interviews after patients receive counselling with OncologIQ Palliative. Several days after the consultation, patients will be asked to participate in an interview. If they agree, they will be interviewed 3–4 weeks after receiving the prognostic information, and additional IC will be collected. The researcher will interview the patients using a structured interview guide, and the interview will be recorded. The interview will then be transcribed word for word. We will conduct these interviews either in person at the outpatient clinic, at the patients’ home, or by telephone, based on the patients’ preferences. The inclusion of patients will start simultaneously with the start of cohort 2.

### Timeline

**Fig. 1:**
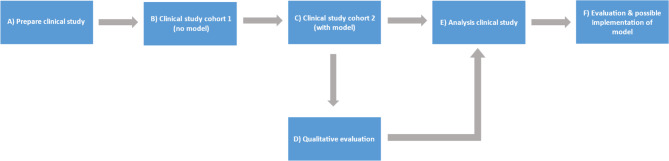
Flowchart illustrating the different study procedures

**Fig. 2:**
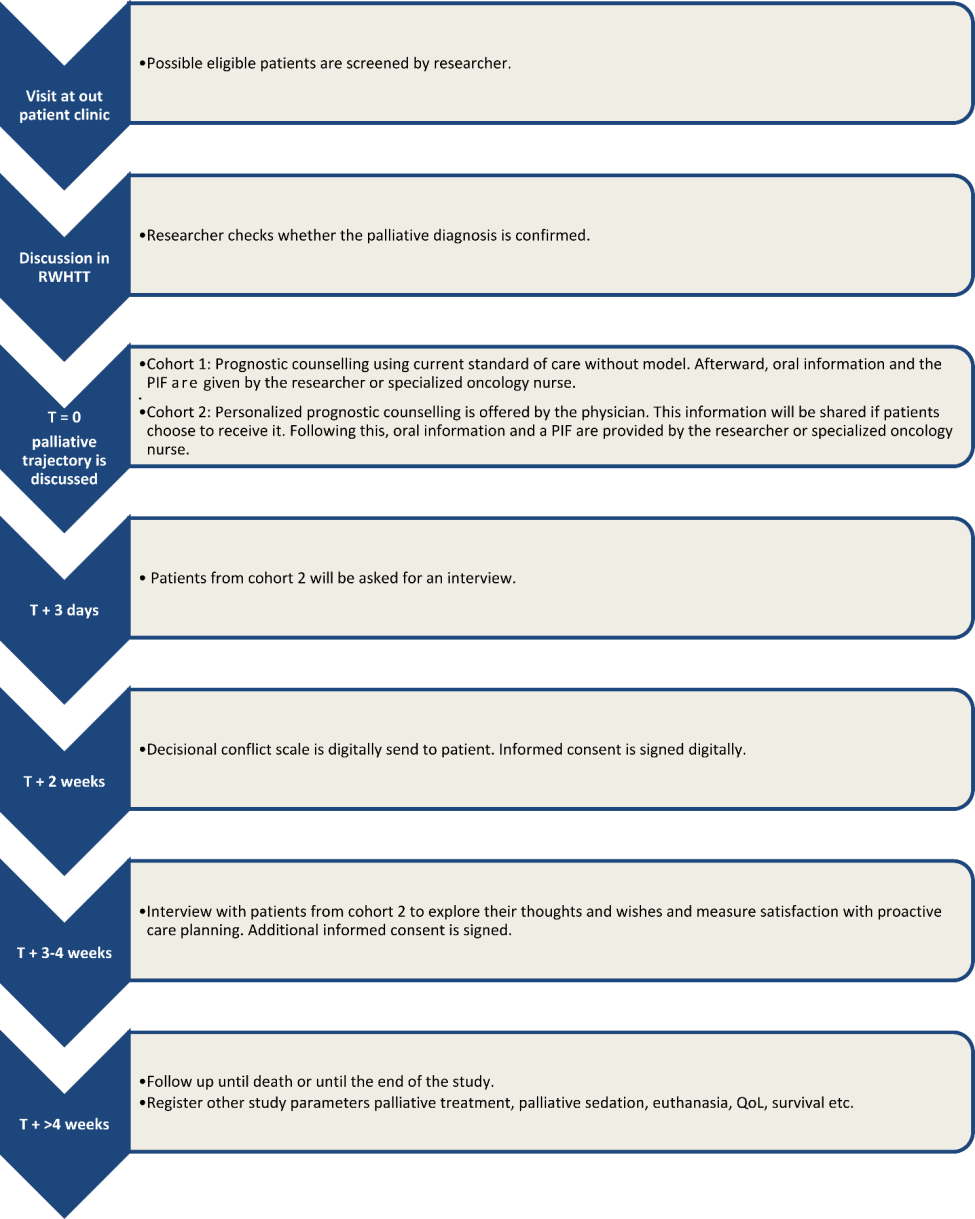
Flowchart illustrating the timeline of study procedures for patients involved in the clinical study and qualitative evaluation

All patients will be actively participating for 1–2 months. During this period, they will complete the EORTC QLQ-C15-PAL questionnaire every 6–8 weeks, the DCS questionnaire 2 weeks after counselling, and possibly participate in an interview 3–4 weeks after counselling. Following this period, patients will no longer be actively involved in the study, as we will gather additional data from the EPF. This data will be collected from every patient over the course of the study (See Figs. 1 and 2 for an overview of the different steps and timeline).

### Outcome evaluation

#### Clinical study

The first outcome measure relates to the amount of decisional conflict and satisfaction with the decision-making process of patients after prognostic counselling. This will be assessed through a comparison of cohort 1 and cohort 2 on decisional conflict scores, measured using the DCS questionnaire (Appendix A) [38, 39]. This questionnaire measures: (a) personal perceptions of uncertainty in choosing options, (b) modifiable factors contributing to uncertainty, such as feeling uninformed, unclear about personal values, and unsupported in decision making, (c) effective decision making, such as feeling the choice is informed, values-based, likely to be implemented, and expressing satisfaction with the choice.

The secondary outcome measures relate to the effect of prognostic counselling on different factors, including: (1) overall survival, (2) palliative treatment, and (3) QoL.

*Overall survival* will be measured from the date of the start of the palliative trajectory until the date of death or the last follow-up visit. Survival outcomes will be compared between both cohorts.

*Palliative treatment* will be assessed by examining whether a patient underwent treatment and the type of treatment received. Data on treatment modalities will be collected for each patient by consulting the EPF.

*Quality of life (QoL)* of patients will be measured using the EORTC QLQ-C15-PAL questionnaire, a shortened version of the EORTC QLQ-C30 [40, 41]. This questionnaire is recommended for use in patients with advanced and incurable cancer with a short life expectancy. The EORTC QLQ-C15-PAL questionnaire consists of 15 questions across 10 domains: physical functioning, emotional functioning, fatigue, pain, dyspnoea, insomnia, appetite loss, constipation, nausea/vomiting, and global health status. In the domains of emotional functioning, physical functioning, and global health status, a higher score indicates a better QoL, while in the other domains, a lower score indicates a better QoL.

### Qualitative evaluation

The primary outcome relates to the effect of prognostic counselling on satisfaction with the decision-making process and proactive care planning. This includes end-of-life discussions on preferred place of death and wishes for palliative sedation and euthanasia. It will be assessed through interviews conducted with patients in cohort 2 after they receive counselling using the model.

### Statistical analysis

#### Clinical study

All values of the variables will be described separately for the control (cohort 1) and intervention group (cohort 2). The values will be expressed as mean ± SD, mean (25th to 75th percentile), or percentage, depending on the variable. Statistical analyses will be performed using the Statistical Package for the Social Sciences and R software. Missing data will be imputed using multiple imputation [42]. 

The DCS is a validated 16-item 5-point Likert-scale measurement for assessing patients’ uncertainty regarding their medical decision. It consists of five subscales measuring: uncertainty, feeling un/informed, values clarity, support and effective decision [39]. The overall score of the DCS ranges from 0 to 100. Higher scores indicate higher decision-related distress. Scores < 25 are associated with implementing decisions, while scores > 37.5 are associated with decision delay or feeling unsure about implementation [39]. Several studies suggest a total score of ≥ 25 as a cut-off for clinically significant decisional conflict [43, 44]. Both total and subscale DCS scores will be calculated in both cohorts. Additionally, the percentage of patients with clinically significant decisional conflict will be calculated.

The differences between control (cohort 1) and intervention group (cohort 2) will be tested by a T-test if the data is normally distributed. If the data is not normally distributed, the Mann-Whitney U test will be used. We will also evaluate the difference in the proportion of patients with clinically significant decisional conflict between cohort 1 and 2 using the chi-square test.

Furthermore, univariable and multivariable analysis will be performed to determine the relationship between e.g. various patient characteristics and the DCS. We will assess the independent contributions of potential influencing factors, such as smoking behaviour, level of education, marital status, age, WHO-performance status, and tumour characteristics, using multivariable regression analysis for the total DCS score and Cox-regression for survival analysis.

The overall survival function in both cohorts will be analysed using the Kaplan-Meier method. Statistical significance will be assessed using the log-rank test. Other secondary outcomes will be analysed using various tests, depending on whether the variable is continuous or categorical and whether it is normally distributed. A p-value of < 0.05 will be considered statistically significant for all analysis.

### Qualitative evaluation

The grounded theory approach will be used to analyse the qualitative data. This implies that the researcher moves back and forth between the population under study and analysis of the data, so that an explanatory theory evolves through an iterative process [45]. All transcripts will be discussed until consensus is reached on the different themes. Themes will be derived from the coded data. These themes will be discussed and if necessary rearranged. When there is no agreement on the themes or on the matching of quotations with the themes, consensus will be reached after an in-depth discussion. The next step will be to verify the results by an additional researcher. NVivo will be used to manage the data.

## Discussion

Providing accurate individual prognostic information to patients with HNSCC in the palliative phase remains challenging. Currently, physicians rely on general numbers and base their estimates on a broad spectrum of HNSCC patients in the palliative phase, complemented by their own experience [22, 23]. However, these predictions often prove inaccurate, as studies indicate a tendency among both physicians and patients to overestimate survival [11, 22–26]. Disclosing more accurate prognostic information could help patients to better prepare for what is to come and assist them in making well-considered end-of-life choices with their loved ones. The prognostic model OncologIQ Palliative enhances predictions and can facilitate more adequate counselling for those requiring such information. This project aims to assess the clinical effect of counselling using this model.

### Challenges and resolutions

Launching this project with the vision to potentially implement OncologIQ Palliative as a future standard of care, we were met with a series of challenges. One such challenge, anticipated during the initial planning phase of the project, was the *short life expectancy and vulnerability* of the patient population. This vulnerability, stemming from their bad condition and issues with vital functions such as breathing and swallowing, as well as their short life span, can make it more difficult for patients to participate in research projects. However, we anticipated that most patients would respond positively to this project, as earlier research shows that patients consider life expectancy a very important topic in the palliative phase and, in the case of a poor prognosis, have a stronger preference for prognostic information presented in a specific quantitative manner. Taking into account this limited time and to minimalize the burden on patients, we are inviting them early in their trajectory to participate. Constantly keeping in mind that we cannot ask much from these patients, we aim to keep the burden for the patients as low as possible. For example, we are digitally sending out the questionnaires and conducting the interviews at their preferred location.

Furthermore, it remains crucial to understand *different individual patient preferences* for prognostic information. Earlier research among HNSCC patients indicated that in the hypothetical case of cancer recurrence and a poor prognosis they tend to express a stronger preference for quantitative individual prognostic information [21]. However, preferences vary widely among all individuals, and not all wish to receive quantitative data about their prognosis. Some may prefer a more qualitative approach, focusing solely on broader aspects of their condition. Another layer of complexity arises from the possible *distinct needs* between patients and their loved ones. While one of them might desire specific numerical probabilities on life expectancy, the other might prefer not to know. Balancing these perspectives could lead to difficulties during consultation sessions. Additionally, *communicating survival probabilities* effectively is essential. However, this can be confusing for patients and their caregivers, as presenting statistical information – such as the individualized chances of survival – requires specific skills.

In conclusion, translating the different individual preferences into effective communication can be complex, and ensuring that patients comprehend these probabilities without causing undue confusion is a delicate task. To address these challenges, we previously conducted interviews with both patients and healthcare professionals to explore those differing topics [34]. By understanding their preferences and refining our communication strategies, we aim to optimize the delivery of this personalized prognostic information during the clinical study.

An unforeseen challenge arose from the unexpected requirement to comply with the Medical Device Regulation (MDR). Previously, it was not standard practice to adhere to *MDR regulations during the research phase*. This mandate added significant complexity to the study design and implementation process, as ensuring compliance with MDR regulations demands additional resources and specialized expertise. While the possibility exists that the final results of our study may not align with our initial expectations, potentially resulting in the model not being implemented as standard care.

Moreover, obtaining a CE marking for the final product poses difficulties. CE marking is essential for ensuring that medical devices comply with European Union (EU) regulations and standards, allowing them to be used within the EU. However, the strict requirements and evaluation processes associated with acquiring a *CE marking can be time-consuming and resource-intensive*. As a result, navigating the regulatory landscape for obtaining a CE marking is a significant operational challenge for our study team. To address these issues, careful consideration and planning were required to ensure compliance with MDR regulations and support the process of obtaining CE marking in the future. Instead of acquiring ethical approval for the entire project at once, we decided to divide our request in two. This was considered necessary to be able to start with the project without any further unnecessary delay. In the meantime, we are engaging with regulatory experts, allocating sufficient resources, and adjusting the study timeline to accommodate to these regulatory requirements.

Despite these challenges, we remain committed and are currently en route to overcome all regulatory hurdles to ensure the successful execution and dissemination of the study results through multiple publications.

## Conclusion

Our results have the potential to lead to the availability of optimal outcome data on the individual life-expectancy of HNSCC patients in the palliative phase. This information can empower physicians in counselling patients by offering more accurate personalized prognostic insights, while also potentially improving patients’ and their loved ones’ understanding and preparedness regarding the situation. By providing a clearer prognosis, we anticipate a shift towards more patient-centred counselling. This could lead to greater patient empowerment, facilitating enhanced end-of-life discussions, and better proactive care planning as they are better prepared. As a result, patients may experience reduced decisional conflict and greater satisfaction with the decision-making process. In addition, we expect that when patients are optimally informed they will choose for less treatment in the palliative phase. All of these aspects can contribute to a better quality of life and quality of care for HNSCC patients in the last phase of their lives. If our findings are proven effective, we are devoted to implementing and offering counselling with OncologIQ Palliative structurally for all our palliative patients who wish to receive this information.

## Electronic supplementary material

Below is the link to the electronic supplementary material.

Supplementary Material 1

Supplementary Material 2

## Data Availability

No datasets were generated or analysed during the current study.

## Abbreviations

ACE-27: Adult Comorbidity Evaluation 27
CE: marking Conformité Européenne marking
cT: Clinical Tumour
cN: Clinical Nodes
cM: Clinical Metastasis
DCS: Decisional Conflict Scale
EPF: Electronic Patient Files
Erasmus MC: Erasmus Medical Centre
EU: European Union
HNSCC: Head and Neck Squamous Cell Cancer
IC: Informed Consent
MDR: Medical device regulation
MEC: Medical Ethical Committee
METC: Medical Ethical Committee (in Dutch: Medisch Ethische Toetsingscommissie)
PROM: Patient Reported Outcome Measure
QoL: Quality of life
RONCDOC: Rotterdam Oncology Documentation
RWHHT: The Rotterdam Multidisciplinary Head and Neck Tumor Board (In Dutch: De Rotterdamse Werkgroep Hoofd-Halstumoren)
SD: Standard deviation
