# Supplementary figures and images for: A study protocol for individualized prognostic counselling in the palliative phase
Source: BMC Palliat Care. 2025 Jan 10;24:9. doi: 10.1186/s12904-025-01647-z (PMC11720302; doi:10.1186/s12904-025-01647-z)

**Appendix A. Decisional Conflict Scale**


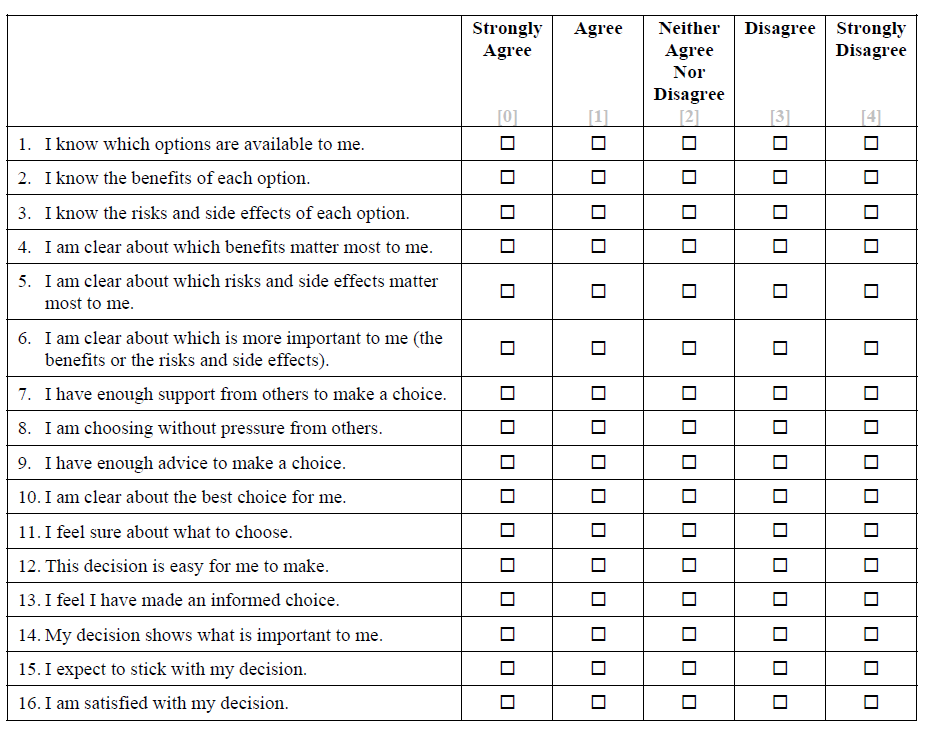

Supplement: Supplementary file 1 — Supplementary Material 1 [file 12904_2025_1647_MOESM1_ESM.docx]
