# Supplementary material for: A study protocol for individualized prognostic counselling in the palliative phase
Source: BMC Palliat Care. 2025 Jan 10;24:9. doi: 10.1186/s12904-025-01647-z (PMC11720302; doi:10.1186/s12904-025-01647-z)
Supplement: Supplementary file 2 — Supplementary Material 2 [file 12904_2025_1647_MOESM2_ESM.pdf]

Erasmus MC
Universitair Medisch Centrum Rotterdam

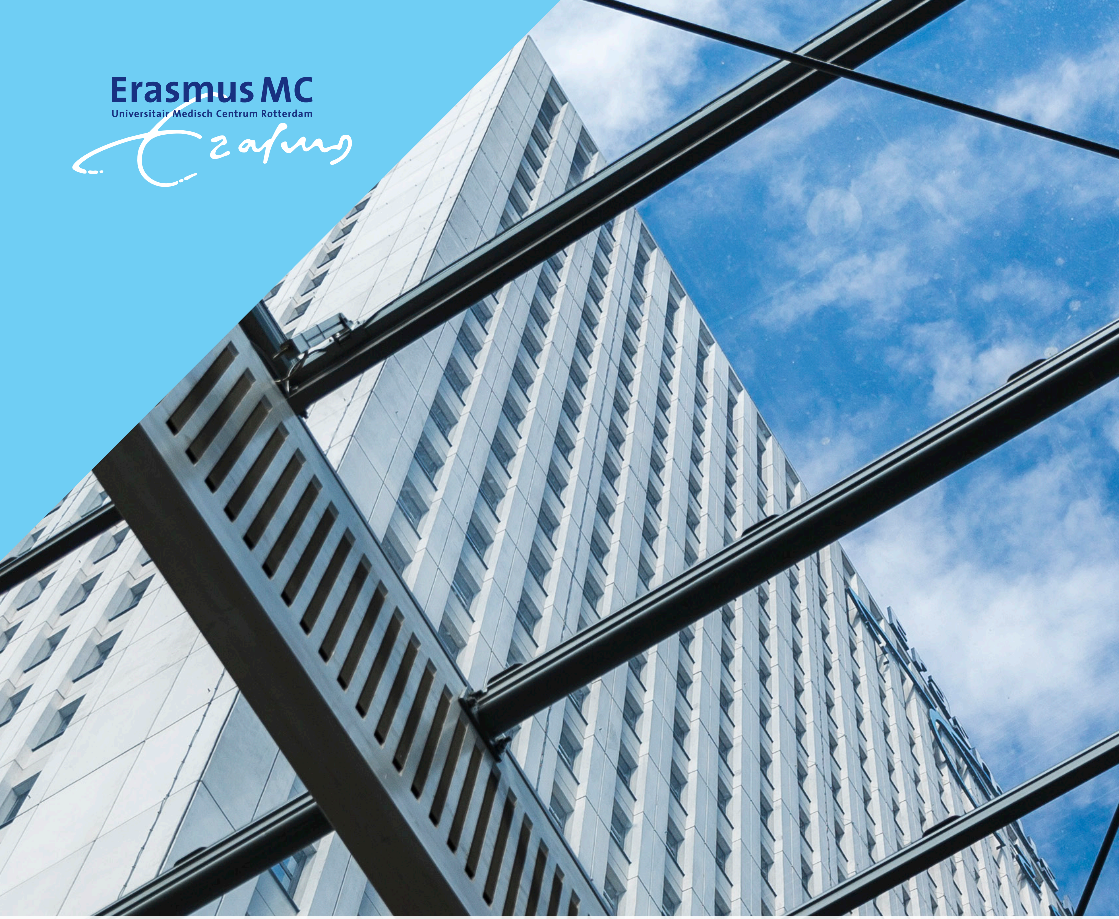

Patiënten informatiebrief

Onderzoek naar de invloed van patiëntenvoorlichting op hoe tevreden hoofd-halskanker patiënten zijn met genomen besluiten in de palliatieve fase[1].

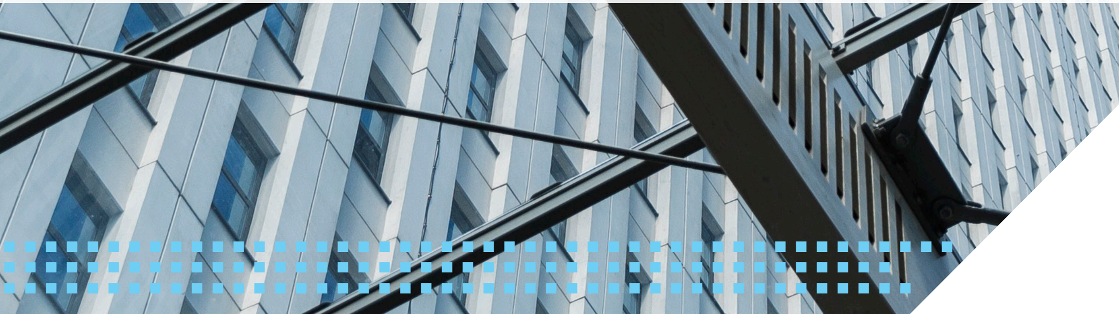

[1] Palliatieve fase betekent dat er geen genezende behandeling meer mogelijk is.

Geachte heer, mevrouw,

U komt in het Erasmus MC vanwege een kwaadaardige aandoening in het hoofd-halsgebied. Zojuist hebben wij u gevraagd deel te nemen aan medisch-wetenschappelijk onderzoek. Dit onderzoek gaat over het verbeteren van de voorlichting die uw behandelend arts u geeft. In totaal vragen wij 160 deelnemers aan het onderzoek deel te nemen. Voordat u beslist om deel te nemen, is het belangrijk om meer te weten over het onderzoek. Leest u deze informatiebrief daarom rustig door. Eventueel kunt u ook meer informatie op http://www.rijksoverheid.nl/mensenonderzoek lezen. Dit is informatie over medischwetenschappelijk onderzoek in het algemeen.

Doel van het onderzoek

Het horen dat er geen mogelijkheid meer is tot genezing van de ziekte of dat u zelf afziet van behandeling, is erg ingrijpend. Het is dan ook van groot belang dat u optimaal geïnformeerd wordt over het beloop van de ziekte. Met dit onderzoek willen wij kijken naar de manier waarop uw behandelend arts u deze voorlichting geeft. Wij willen onderzoeken hoe tevreden u bent met de gegeven voorlichting en of deze manier van voorlichting invloed heeft op de besluiten die u neemt. Het gaat om de besluiten die u neemt rondom behandelwensen en zorgplanning in de laatste levensfase. Wij willen ook kijken of dit invloed heeft op uw kwaliteit van leven.

Hoe wordt het onderzoek uitgevoerd?

Na het gesprek met de verpleegkundig consulent, vragen wij u om eenmalig één vragenlijst in te vullen (in totaal 16 vragen). Wij vragen u om dit 2 weken na het voorlichtingsgesprek met de arts te doen. Deze vragenlijst gaat over hoe tevreden u bent met uw genomen besluiten. Het gaat om de besluiten die u neemt rondom behandelwensen en zorgplanning in de laatste levensfase. Mogelijk kunt u ook één keer gevraagd worden voor een aanvullend interview.

Wat wordt er van u verwacht?

Als u besluit deel te nemen aan deze studie, vragen wij u om de gemaakte afspraken na te komen en de instructies zo goed mogelijk op te volgen.

Wat is anders dan de standaard behandeling die u krijgt?

Door uw deelname aan het onderzoek verandert er niets aan de normale voorlichting die u krijgt. Wij vragen u om 2 weken na het gesprek met de arts eenmalig één extra vragenlijst in te vullen. Dit kunt u gewoon thuis via de computer doen. Daarnaast kunt u mogelijk ook eenmalig gevraagd worden voor een aanvullend interview.

Wat zijn de mogelijke voor- en nadelen van deelname aan dit onderzoek?

U hebt zelf geen direct voordeel van deelname aan dit onderzoek. Voor de toekomst kan het onderzoek voordeel opleveren voor de voorlichting aan toekomstige patiënten.
Mogelijke nadelen van deelname kunnen zijn:

- Het invullen van de vragenlijst kost u gemiddeld vijf tot tien minuten.
- Als u gevraagd wordt voor het interview en u ervoor kiest om daaraan deel te nemen, kost dit u ongeveer één uur.

Wat gebeurt er als u niet wenst deel te nemen aan dit onderzoek?

U beslist zelf of u meedoet aan het onderzoek. Deelname is geheel vrijwillig. Als u besluit niet mee te doen, hoeft u verder niets te doen. U hoeft ook niet te zeggen waarom u niet mee wilt doen. Indien u niet deelneemt aan het onderzoek, verandert er niets aan de normale voorlichting en behandeling die u krijgt.

Wat gebeurt er als het onderzoek is afgelopen?

Na afloop van het onderzoek kunt u –indien u dat wenst- een overzicht van de resultaten krijgen.
U kunt op ieder moment stoppen met deelname aan dit onderzoek wanneer u dat wenst.

Wat gebeurt er met uw gegevens?

We verzamelen, gebruiken en bewaren uw gegevens om de vragen van dit onderzoek te kunnen beantwoorden. Om uw privacy te beschermen geven wij uw gegevens een code. Als we uw gegevens verwerken, gebruiken we steeds alleen die code. Sommige personen kunnen wel uw naam en andere persoonlijke gegevens zonder code inzien. Dit zijn mensen die controleren of de onderzoekers het onderzoek goed en betrouwbaar uitvoeren. Deze personen houden uw gegevens geheim. Wij vragen u voor deze inzage toestemming te geven. Wij bewaren uw gegevens 15 jaar in het ziekenhuis. Daarvoor geeft u toestemming als u meedoet aan dit onderzoek. Als u dat niet wilt, dan respecteren wij uw keuze.

Welke medisch-ethische commissie heeft dit onderzoek goedgekeurd?

Voor dit onderzoek is goedkeuring verkregen van de Medisch Ethische Toetsingscommissie Erasmus MC. De voor dit onderzoek geldende internationale richtlijnen zullen nauwkeurig in acht worden genomen.

Erasmus MC
Universitair Medisch Centrum Rotterdam

Wilt u verder nog iets weten?

Vragen over het onderzoek kunt u stellen aan de arts-onderzoeker. Zie hieronder de contactinformatie.

Contactgegevens:

Drs. B.N. van den Besselaar, Afdeling KNO/ Hoofd Hals Chirurgie, arts-onderzoeker, **06-38 82 00 25**
Dr. M.P.J. Offerman, Afdeling KNO/ Hoofd Hals Chirurgie, hoofdonderzoeker.

Buiten kantooruren:

U contact opnemen met de telefoniste van het Erasmus MC **(010-7040704)** en vragen naar de dienstdoende KNO-arts (24 uur per dag bereikbaar wanneer nodig).

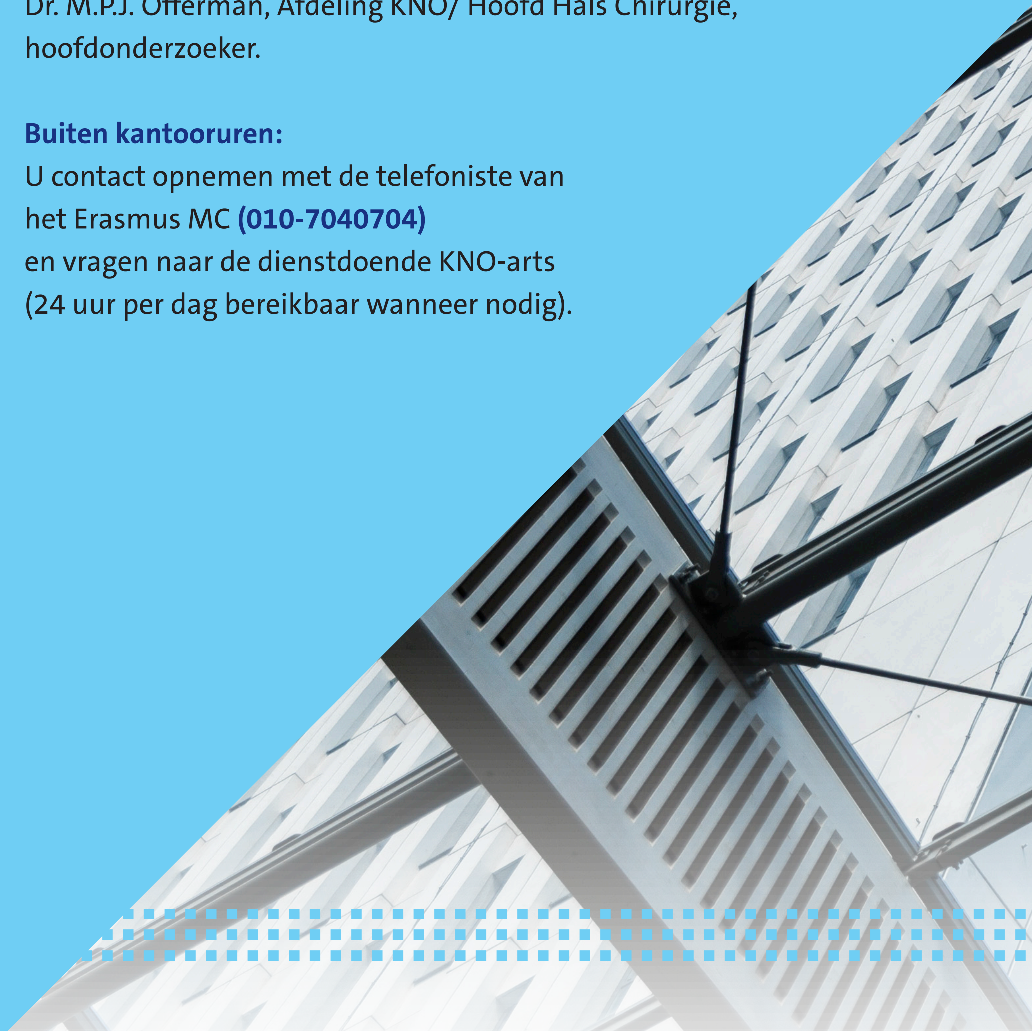

ERA220262
